# Incidental Finding of an Inflammatory Myofibroblastic Tumor of the Urinary Bladder: A Case Report

**DOI:** 10.7759/cureus.90006

**Published:** 2025-08-13

**Authors:** Panagiotis A Panagopoulos, Konstantinos Douroumis, Penelope Korkolopoulou, Angeliki Papachristou, Afroditi Nonni, Dionysios Mitropoulos, Napoleon Moulavasilis, Evangelos Fragkiadis

**Affiliations:** 1 First Department of Urology, National and Kapodistrian University of Athens (NKUA) School of Medicine, Athens, GRC; 2 First Department of Pathology, National and Kapodistrian University of Athens (NKUA) School of Medicine, Athens, GRC; 3 First Department of Urology, Laiko General Hospital, National and Kapodistrian University of Athens (NKUA), Athens, GRC; 4 First Department of Urology, National and Kapodistrian University of Athens, Athens, GRC

**Keywords:** alk, imts, inflammatory myofibroblastic tumor, sma, urinary bladder

## Abstract

Bladder inflammatory myofibroblastic tumors (IMTs) are rare lesions with intermediate malignant potential and unclear management guidelines. We report the case of a 61-year-old male presenting with lower urinary tract symptoms and a bladder mass. Histology revealed spindle cells with mild atypia and chronic inflammation, and anaplastic lymphoma kinase (ALK) positivity suggested a TPM3-ALK fusion. The tumor was completely resected via transurethral resection of the bladder tumor (TURBT), with no recurrence at the six-month follow-up. This case illustrates the diagnostic complexity of bladder IMTs and emphasizes the importance of histopathology and close surveillance due to the risk of recurrence and rare metastasis.

## Introduction

Inflammatory myofibroblastic tumors (IMTs) are rare mesenchymal tumors with intermediate biological potential. Although they tend to recur, metastasis is uncommon [1,2]. These tumors consist of myofibroblastic spindle cells mixed with an inflammatory infiltrate, which includes plasma cells, lymphocytes, and eosinophils. While IMTs are most commonly found in the abdominal cavity and lungs, they have also been reported in other areas, such as the head and neck [3,4].

Bladder IMTs are extremely rare, accounting for less than 1% of all bladder tumors. The most common symptom is hematuria, but some patients may also present with lower urinary tract symptoms (LUTS), abdominal pain, or even an incidental bladder mass [5]. When examined macroscopically, these tumors often appear as polypoid masses or submucosal nodules. Their immunohistochemical profile can vary; some studies have shown positivity for actins and vimentin, while p53 staining is typically weak or absent. However, actin expression is not dependable for distinguishing between benign and malignant tumors, as α-smooth muscle actin positivity has been observed in 63% of IMTs and 43% of sarcomas.

One of the more promising markers in bladder IMTs is the presence of cytoplasmic immunostaining for anaplastic lymphoma kinase (ALK), which has been found in 8% to 89% of cases. ALK is a tyrosine kinase receptor and part of the insulin-like growth factor receptor superfamily. Alterations in the ALK gene are seen in several mesenchymal tumors, and since ALK is generally not expressed in normal fibroblasts, its activation and overexpression could play a significant role in the development of these tumors [6].

We present a case of incidental diagnosis of an IMT of the urinary bladder. These cases generally present a diagnostic challenge for the pathologist, along with challenges in treatment and follow-up decisions for urologists, as they represent a rare entity not discussed in the urologic guidelines. With this case, we aim to present the patient’s path to diagnosis, treatment, and follow-up, and to contribute to the current sparse literature on this type of tumor.

## Case presentation

**Figure 1:**
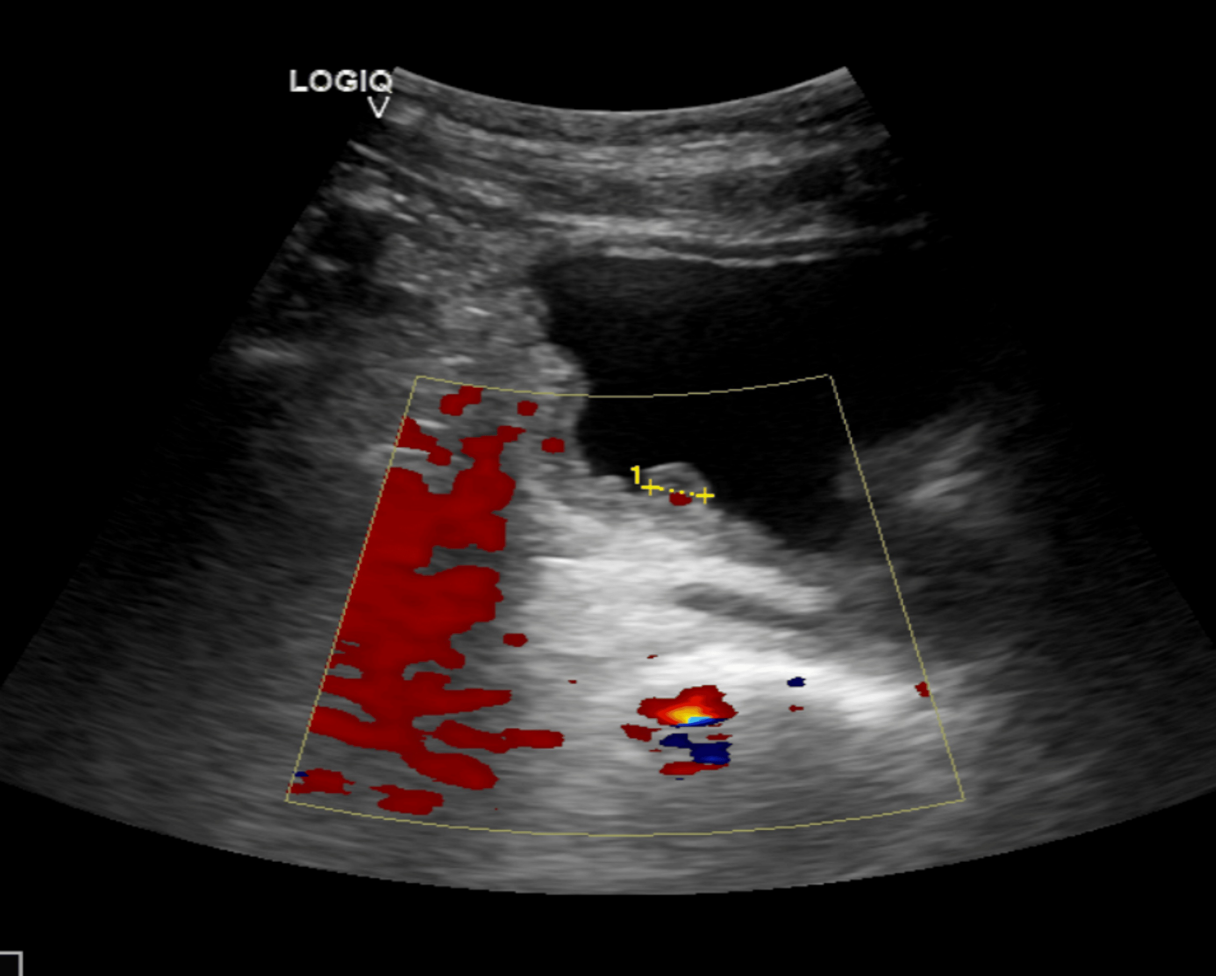
Ultrasound of the bladder. Doppler imaging showing internal vascularity of the 2.5 cm³ lesion.

A 61-year-old male patient presented to the outpatient department of our clinic in July 2024, complaining of LUTS for the past three weeks. No significant medical history was reported, other than two episodes of syphilis of the external genitalia. The physical examination was unremarkable. Further laboratory tests, including urine analysis, urine cytology, and ultrasound, were ordered, and re-evaluation of the patient was scheduled. The ultrasound revealed a 2.5 × 1.5 cm³ polypoid mass in the bladder, with evidence of internal vascularity on Doppler imaging (Figure 1).

Urine analysis and cytology results were normal. These findings suggested a bladder tumor; therefore, transurethral resection of the bladder tumor (TURBT) was performed. Intraoperatively, a single exophytic papillary tumor of the left bladder wall was observed. The tumor was excised entirely. As the tumor was deemed low grade, no deep sections of the tumor bed were obtained. Postoperatively, a single instillation of epirubicin was administered on the day of the TURBT. The patient had a smooth recovery and was discharged on the first postoperative day, after catheter removal.

The pathology report revealed a neoplastic lesion within the bladder wall, with low-to-moderate cellularity, composed of spindle-shaped cells within a loose stromal matrix. The tumor cells exhibited mild atypia and low mitotic activity. A characteristic finding was the presence of chronic inflammatory elements and eosinophils between the neoplastic cells. The overlying urothelium was hyperplastic over a considerable extent, with compressive alterations and mild atypia that could not be identified as true atypia.

Immunohistochemical analysis revealed the following profile:

ALK (clone D5F3): Strong, diffuse cytoplasmic positivity; Desmin: Focally positive (+); Calponin: Weakly positive (+); CK8/18: Partially positive (+); CK20: Negative (-); CD34: Negative (-); SMA: Positive (+); Ki67: 10% in tumor cells, <10% in epithelial cells.

**Figure 2:**
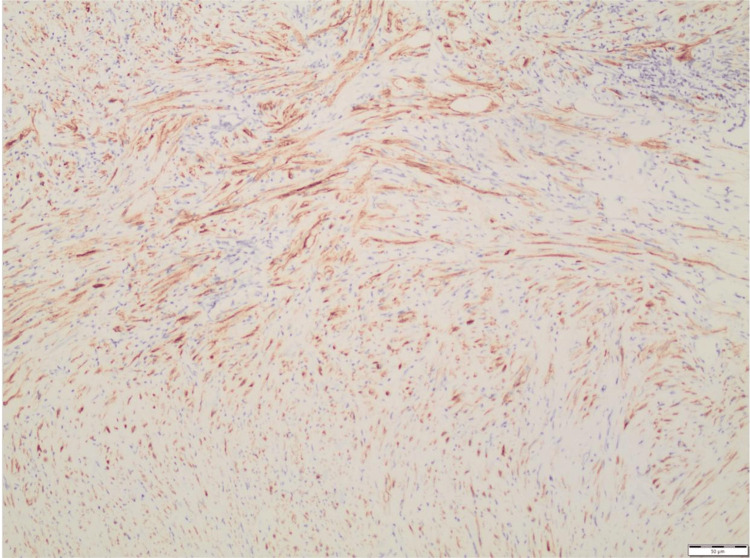
Anaplastic lymphoma kinase. Strong, diffuse cytoplasmic positivity for ALK was observed.

**Figure 3:**
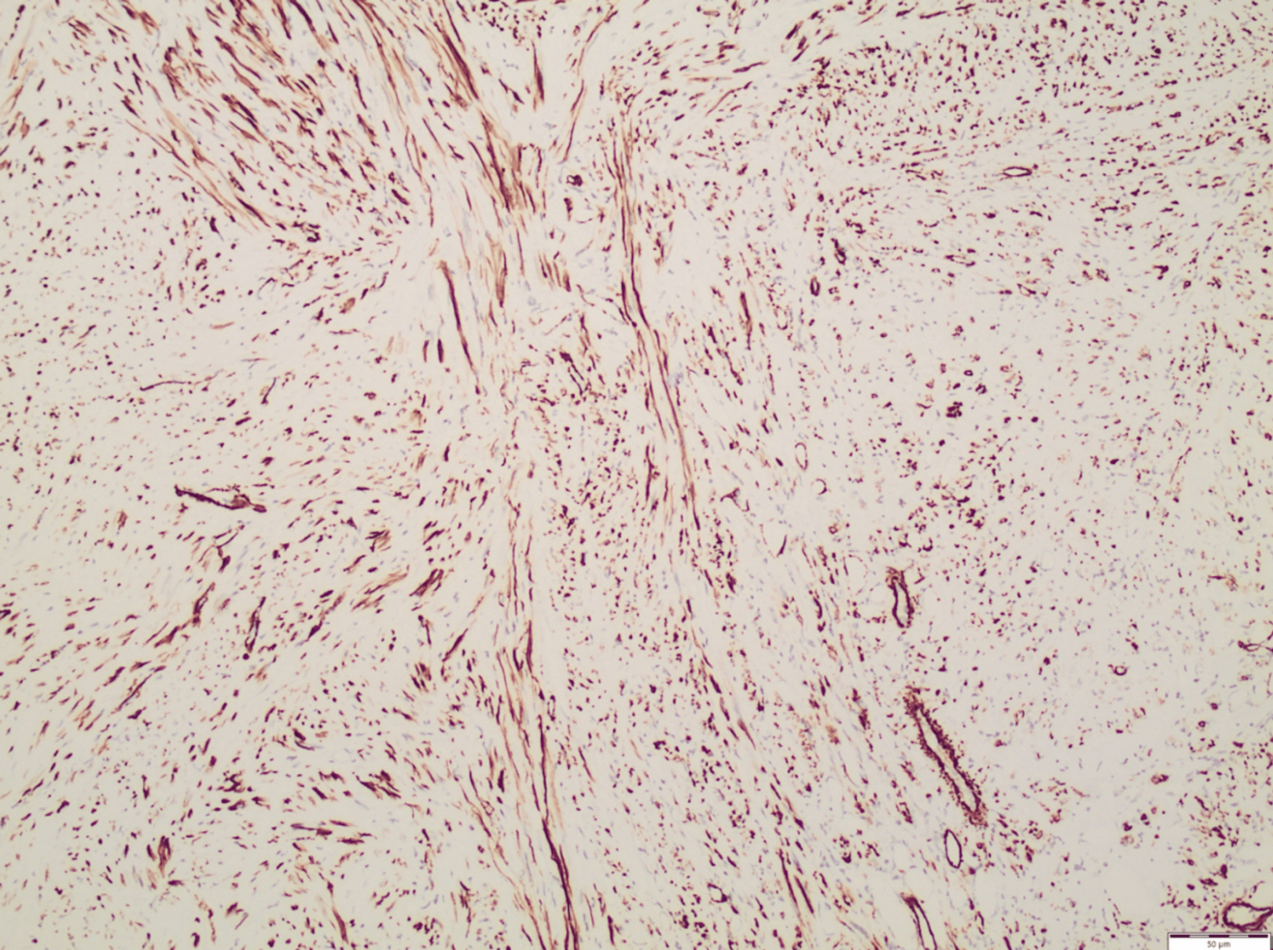
Smooth muscle actin (SMA). SMA staining of the specimen was positive.

The above findings led to the conclusion that the tumor represented a mesenchymal lesion of the bladder wall with immunomorphological characteristics consistent with IMT. The ALK immunostaining pattern suggested a possible TPM3-ALK fusion (Figures 2-3).

**Figure 4:**
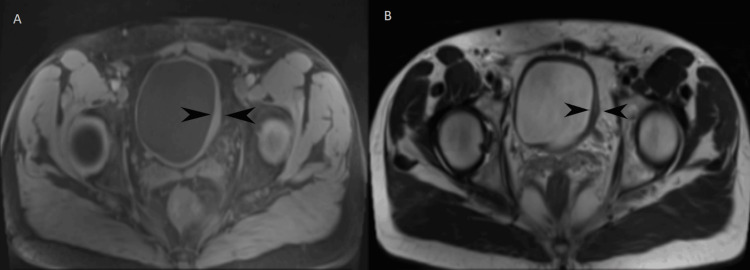
MRI of the lower abdomen one month post-surgery. A: T1-weighted imaging; B: T2-weighted imaging. The only finding was thickening of the bladder wall, likely due to postoperative alterations.

**Figure 5:**
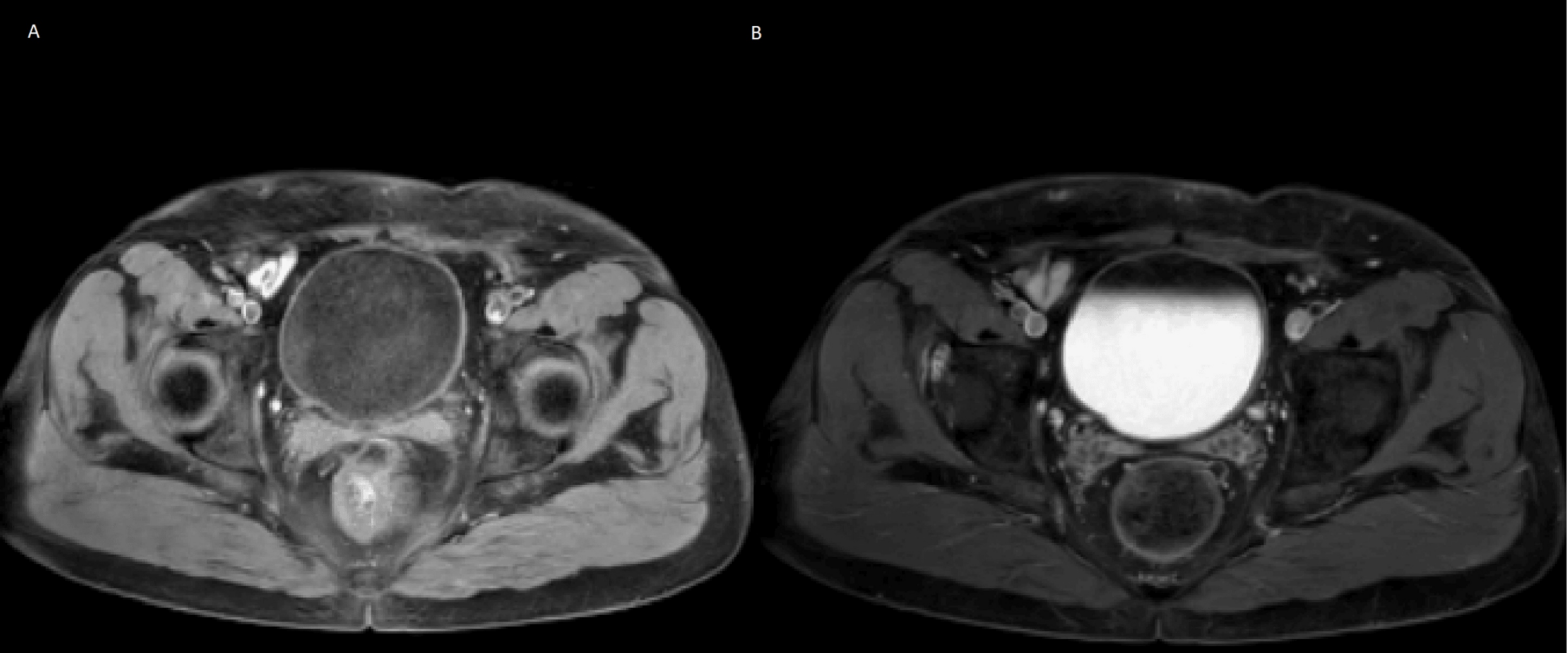
Magnetic resonance imaging of the lower abdomen. A: T1-weighted imaging; B: T1-weighted imaging with contrast enhancement. No remarkable findings were observed.

Following the pathology report, the case was discussed in a multidisciplinary tumor board consisting of urologists, oncologists, and radiation oncologists. As the literature on this entity is limited, a follow-up strategy consisting of cystoscopy and imaging was recommended. Cystoscopy was performed at three and six months post-surgery, both of which were normal. MRI of the abdomen was conducted one month after surgery for local tumor staging, which showed only a thickened bladder wall, likely due to postoperative changes (Figure 4). A follow-up MRI at six months was normal (Figure 5). The patient is currently under follow-up, consisting of cystoscopy and ultrasound every six months. This decision was based on the intermediate malignant potential of IMTs.

## Discussion

Bladder IMT is among the rarest urinary tumors. The available literature does not provide sufficient data on the optimal diagnosis, management, and follow-up protocol [7]. It has been identified in various organs, including the lungs, mesentery, retroperitoneum, and abdominal soft tissue. In the genitourinary tract, it can occur in the kidneys, ureter, testis, and bladder [8].

Macroscopic hematuria is the most frequently reported symptom. Approximately 40% of cases are initially diagnosed due to other LUTS [5]. Our case presented with LUTS. In such cases, carcinoma in situ of the bladder should be suspected, and urinary cytology should be included in the diagnostic workup [9]. The urinary cytology of our patient was negative; however, cases in the literature have been described where cytology has shown remarkable findings [10]. There are no radiologic characteristics that allow differentiation of these tumors from malignant urothelial carcinoma. As a result, the pathology report is crucial for proper diagnosis [11].

Therapeutic options include partial cystectomy and TURBT, with TURBT being performed in more than 50% of cases, based on a systematic review of 75 cases [7]. Partial cystectomy is considered a safe option, as IMTs do not have an increased risk of recurrence or metastasis. However, in cases of muscle-invasive disease, radical cystectomy should be performed. The decision to perform TURBT or partial cystectomy depends on the clinical status of the patient and the size of the tumor. At present, there are no specific guidelines favoring one approach over the other. Partial cystectomy may also be performed using a laparoscopic or robot-assisted approach [12]. In our patient, we opted for TURBT, as the tumor was fully removed, it was non-muscle invasive, and IMTs generally have an excellent prognosis.

From a pathological perspective, the most common subtype of bladder IMT is the spindle cell pattern, present in more than 50% of cases. Other subtypes reported in IMTs include the myxoid or vascular pattern and the fibrous hypocellular pattern. Up to 40% of these tumors have mixed histological characteristics [13].

The immunohistochemical profile of these tumors typically includes positive staining for SMA and variable positivity for desmin. ALK positivity has also been reported in approximately 70% of bladder IMTs [7]. The notable frequency of ALK expression has made ALK a high-value marker for diagnosing IMTs. ALK fusion proteins include TPM3-ALK. The minimal cytologic atypia observed in IMTs is a key feature that distinguishes them from other mesenchymal malignancies, such as leiomyosarcoma and sarcomatoid carcinoma [14]. ALK-positive tumors are associated with a higher recurrence rate, whereas ALK-negative tumors tend to be more locally advanced and may occasionally metastasize [4].

The overall long-term outlook for bladder IMTs is favorable. Reported recurrence rates range between 9% and 21%, while metastasis occurs in approximately 4% of cases [7]. It is important to acknowledge that IMTs have the potential to metastasize, emphasizing the need for extended follow-up monitoring. Regular cystoscopies and computed tomography or MRI scans should be performed. Further guidelines should be established for the management and follow-up of these tumors. As the current literature is scarce, a standardized follow-up protocol cannot yet be established. The most recent systematic review found that different authors employed varying follow-up protocols [7]. ALK expression could potentially serve as a useful index to tailor the follow-up strategy, as ALK-positive tumors tend to recur more frequently, making them suitable candidates for more regular cystoscopic examinations [4].

## Conclusions

Bladder IMTs are rare lesions with intermediate malignant potential. Their distinction from bladder cancer at initial presentation is almost impossible, and therefore TURBT should be the initial management option. Diagnosis is based on pathology, although the immunohistochemical profile can be highly variable. This case highlights that a complete TURBT is sufficient in most cases for this type of tumor, which aligns with findings from the literature. As this entity is very rare, an individualized follow-up protocol should be implemented, consisting of both cystoscopy and imaging. With this case, we hope to contribute to the current knowledge about this rare tumor and to highlight the importance of a complete initial TURBT in its management.
